# Thirteen draft genome assemblies of *Bacillus* spp. isolated from HLB-impacted citrus trees

**DOI:** 10.1128/mra.00602-24

**Published:** 2024-09-10

**Authors:** Flávia Campos Vieira, Alex Blacutt, Christopher Drozd, Polrit Viravathana, Nichole Ginnan, M. Caroline Roper

**Affiliations:** 1 Department of Microbiology and Plant Pathology, University of California, Riverside, California, USA; University of Strathclyde, Glasgow, United Kingdom

**Keywords:** citrus microbiome, *Bacillus*, Huanglongbing, whole-genome sequencing

## Abstract

We report the draft genome assembly, annotation, and phylogenetic placement of 13 *Bacillus* spp. isolates isolated from citrus groves under high (Florida) or low (California) Huanglongbing disease pressure.

## ANNOUNCEMENT

*Bacillus* is a well-studied genus of Gram-positive, spore-forming bacteria, commonly soil-borne and plant-associated (1–3). Many *Bacillus* species produce a range of secondary metabolites and volatile organic compounds that are beneficial to plants through various mechanisms, such as pathogen suppression or plant growth promotion (4–7). In this study, we announce the draft genome assemblies of thirteen *Bacillus* spp. isolated from citrus trees in areas of high (Florida) or low (California) Huanglongbing disease pressure.

Sampling and bacterial isolation were previously described in a bioprospecting effort to generate a citrus-associated microbial repository (8). Leaves, stems, and roots were collected from citrus orchards in Florida and California, detailed in Table 1. Samples were macerated with 1X PBS and propagated on tryptic soy agar (TSA) and potato dextrose agar with 0.1 g/liter tetracycline hydrochloride (PDA) and incubated at 28°C for 4 days. The microbial consortia on each plate were scraped after adding 1 mL of 1X PBS to generate bulk culture tubes. Isolates were recovered from bulk culture tubes on TSA and PDA and incubated at 28°C for up to 5 days. Single colonies of each bacterial isolate were streaked to purity, and cryostocks of each isolate were stored in 15% glycerol at −80°C.

**TABLE 1:** Genome assembly statistics

Isolates	Species	Location (city, state)	Tree no	Tissue of origin	Raw reads	No of contigs	N50 (bp)	Completeness	GC content (%)	Total size (bp)	Predicted gene no	Ani (%)	Accession no of ref. genome	SRA accession no	WGS accession no
CB00019	*Bacillus velezensis*	Riverside, California	T1	Stem	12,000,690	22	1,085,110	99.41	45.84	4,198,490	4,238	98.27	GCF_001461825.1	SRR26973678	JAXKIA000000000
CB00021	*Bacillus velezensis*	Riverside, California	T1	Stem	10,508,320	22	1,085,110	99.41	45.84	4,198,340	4,235	98.27	GCF_001461825.1	SRR26973677	JAXKIB000000000
CB00027	*Bacillus velezensis*	Riverside, California	T1	Rootstock	13,949,264	23	1,085,110	99.41	45.84	4,195,249	4,238	98.27	GCF_001461825.1	SRR26973673	JAXKIC000000000
CB00069	*Bacillus pumilus*	Orlando, Florida	T8	Leaf	13,465,950	64	151,282	97.28	40.88	3,906,740	4,104	95.53	GCF_900186955.1	SRR26973672	JAXKID000000000
CB00676	*Bacillus cereus*	Clewiston, Florida	T51	Stem	3,236,434	98	186,813	99.43	34.95	5,551,588	5,644	97.22	GCF_006384875.1	SRR26973671	JAXKIE000000000
CB00687	*Bacillus velezensis*	Fort Pierce, Florida	T19	Leaf	2,631,398	17	1,008,008	99.33	46.50	4,092,183	4,128	98.27	GCF_001461825.1	SRR26973670	JAXKIF000000000
CB00729	*Bacillus safensis*	Clewiston, Florida	T50	Leaf	2,560,758	22	871,191	99.41	41.50	3,677,118	3,763	97.4	GCF_000691165.1	SRR26973669	JAXKIG000000000
CB00742	*Bacillus subtilis*	Orlando, Florida	T17	Leaf	2,845,570	39	306,880	97.35	43.5	4,219,379	4,450	98.27	GCF_000009045.1	SRR26973668	JAXKIH000000000
CB00893	*Bacillus subtilis*	Orlando, Florida	T17	Leaf	5,694,468	44	271,969	97.35	43.5	4,215,674	4,453	98.27	GCF_000009045.1	SRR26973667	JAXKII000000000
CB00902	*Bacillus velezensis*	Clewiston, Florida	T36	Roots	3,495,930	43	227,074	99.41	46.07	4,061,377	4,090	98.25	GCF_001461825.1	SRR26973666	JAXKIJ000000000
CB00904	*Bacillus velezensis*	Clewiston, Florida	T31	Leaf	3,654,122	48	225,439	99.41	46.07	4,061,111	4,095	98.25	GCF_001461825.1	SRR26973676	JAXKIK000000000
CB00909	*Bacillus velezensis*	Clewiston, Florida	T31	Leaf	3,303,372	38	234,435	99.41	46.08	4,063,280	4,086	98.25	GCF_001461825.1	SRR26973675	JAXKIL000000000
CB00912	*Bacillus velezensis*	Clewiston, Florida	T31	Leaf	3,614,876	42	234,440	99.41	46.07	4,063,583	4,093	98.25	GCF_001461825.1	SRR26973674	JAXKIM000000000

**Fig 1:**
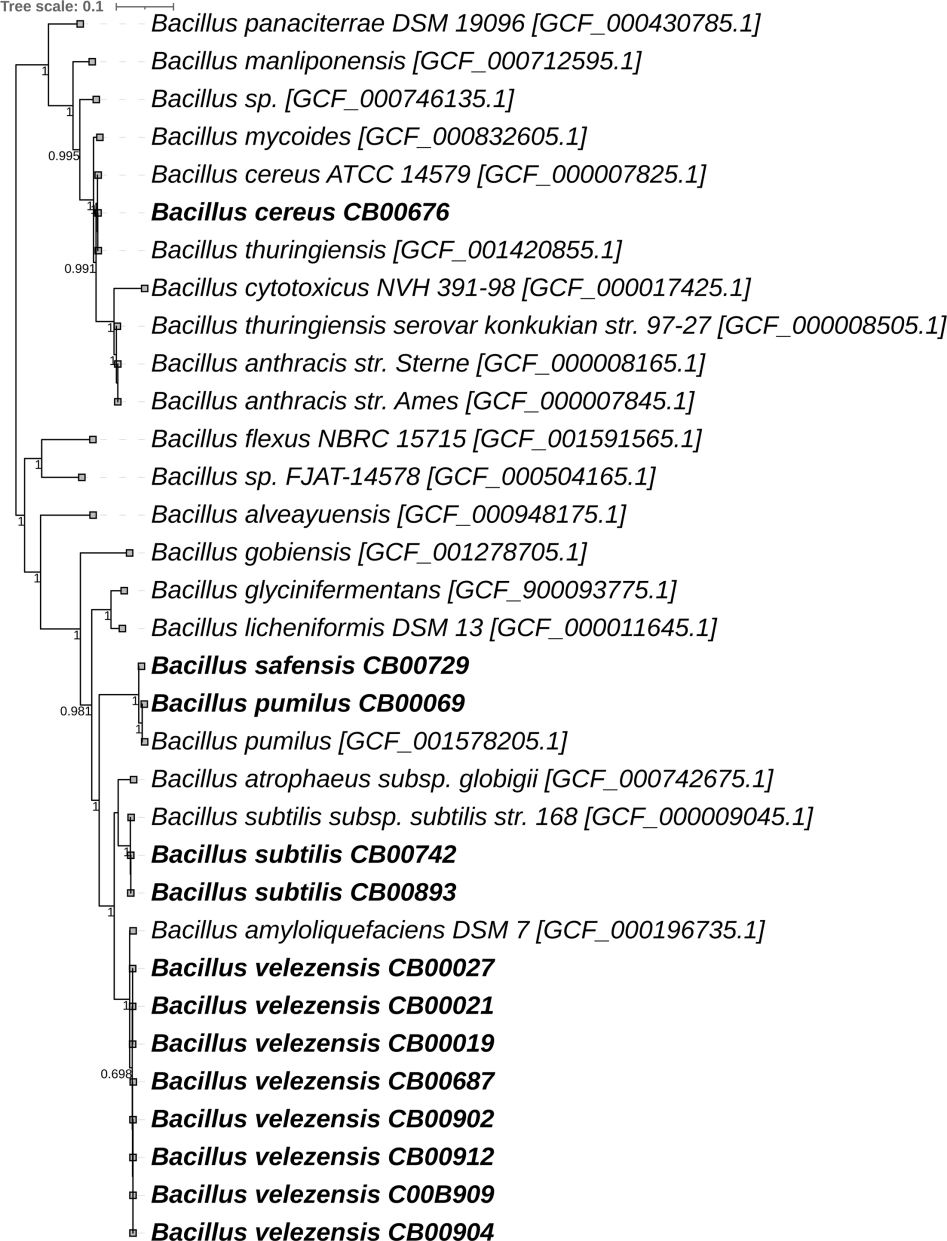
Phylogenetic tree of *Bacillus*, constructed on the SpeciesTree (v2.2.0) app of Kbase, using a set of 49 core, universal genes defined by COG (clusters of orthologous groups) gene families. The set of query genomes is inserted into curated multiple sequence alignment (MSA) for each COG family; the MSAs are concatenated, and a phylogenetic tree is reconstructed using FastTree2 (v2.1.10) (fastest setting) to infer approximately maximum likelihood. The bootstrap scores are shown on each node. Species marked in bold are described in this announcement. The closely related genomes used to build the tree are labeled with the NCBI RefSeq species name and between brackets the GCF identifiers.

DNA was extracted from a single colony of each isolate grown overnight at 28°C in tryptic soy broth, using the Wizard Genomic DNA Purification Kit (Promega Corporation, Madison, Wisconsin). Library preparation and sequencing were performed by SeqCenter (Pittsburgh, Pennsylvania). Libraries were prepared using the Illumina DNA Prep kit and IDT 10 bp unique dual indexing (UDI) indices and sequenced on an Illumina NovaSeq 6000, producing 151-bp paired-end reads. Demultiplexing, quality control, and adapter trimming were performed with bcl-convert (v4.0.3) (9). The total number of paired-end raw reads ranged from 2,560,758 to 13,949,264. Sequence analysis from quality control to annotation was performed on the KBase web service (10), and the publicly available narrative containing analyses and data can be found at https://doi.org/10.25982/157793.280/2368552 (11). Default parameters were used, except where noted. Quality of raw reads was assessed using FastQC (v0.12.1) (12), and quality control was performed using JGI RQCFilter pipeline BBTools (v38.22) (13) and PRINSEQ (v0.20.4) (14). Genomes were assembled using SPAdes (v3.15.3) (15) with k-mer sizes set for 21, 33, 55, 77, 91, and 111, and quality of assemblies was assessed with QUAST (v4.4) (16) and CheckM (v1.0.18) (17). Contigs in the resulting assemblies ranged from 17 to 98, and the N50 value ranged from 151,282 to 1,085,110. The completeness of the genomes was between 97.28 and 99.41 and the GC content percentage from 34.95 to 46.50. Genomes were annotated using Prokka (v1.14.5) (18), revealing the largest genome size of 5,551,588 bp from *Bacillus cereus* and the smallest genome size of 3,677,118 bp from *B. safensis*. The number of predicted genes ranged from 3,763 to 5,644 (Table 1). Taxonomic identification was performed on GTDB-Tk (v2.3.2) (19), utilizing FastANI (20). A *Bacillus* phylogenetic tree with closely related species was constructed using FastTree2 (v2.1.11) (21) through SpeciesTree (v2.2.0) on KBase and annotated on iTOL (v6.8.1) (22) (Fig. 1).

## Data Availability

The accession number for the raw reads and whole-genome sequences of the isolates are described in Table 1. The Sequence Read Archive and genomic data have been deposited in GenBank BioProject under no. PRJNA1046128. Analyses are available through the Kbase narrative: https://doi.org/10.25982/157793.280/2368552.
